# Recent advances in the utilization of polyaniline in protein detection: a short review

**DOI:** 10.1039/d2ra05893f

**Published:** 2022-11-16

**Authors:** Ufana Riaz, Nuzhat Nabi, India Pointer, Amit Kumar, Darlene. K. Taylor

**Affiliations:** Materials Research Laboratory, Department of Chemistry, Jamia Millia Islamia New Delhi-110025 India uriaz@jmi.ac.in ufana2002@yahoo.co.in; Department of Chemistry and Biochemistry, North Carolina Central University NC 27707 USA; Theory & Simulation Laboratory, Department of Chemistry, Jamia Millia Islamia New Delhi-110025 India akumar1@jmi.ac.in

## Abstract

Various reports have been published based on covalently attaching biomolecules to polyaniline (PANI). The functional groups connected to the surface of polymeric units determine the immobilization method as well as the method of detection. The present mini-review aims at covering recent advances in the field of protein binding and detection using PANI. Several proteins have been attached to the polymer using different immobilization techniques. The application of PANI in protein detection has also been discussed along with the future scope of these materials in diagnosis and detection.

## Abstract 2

Attachment of biomolecules to polyaniline.

## Graphical abstract

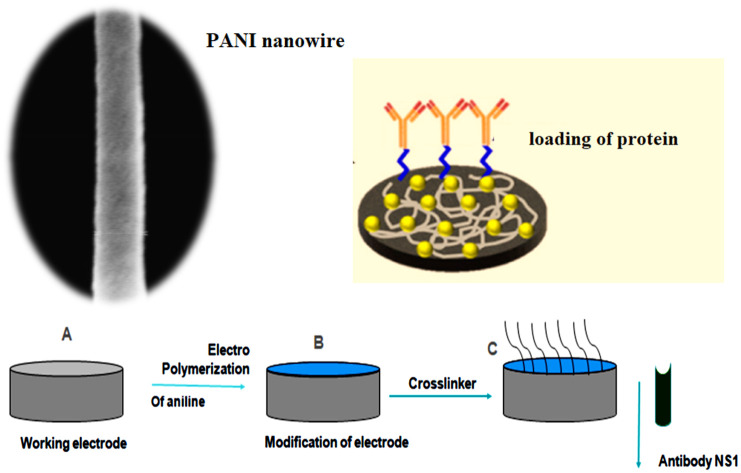

## 1. Introduction

**Fig. 1:**
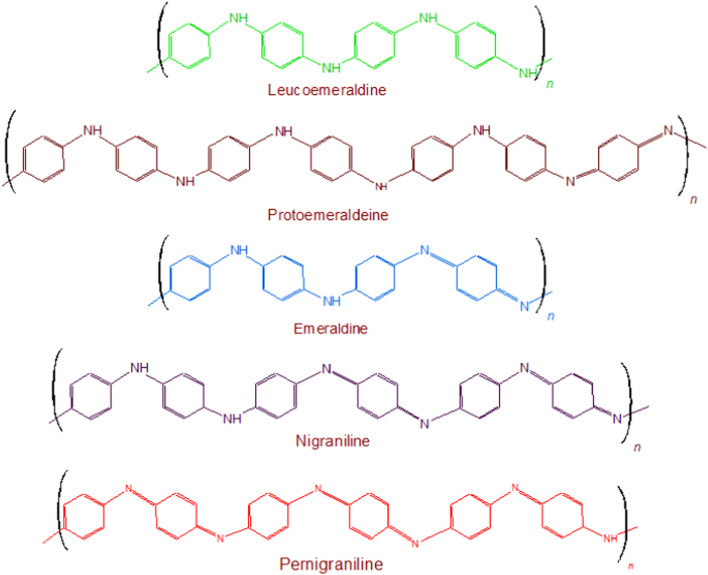
Different redox states of PANI.

Polyaniline (PANI) was discovered during the late 19th century, as a semi-flexible conducting polymer, which established itself as one of the versatile materials in all well-known areas of science and technology including the electrochromic devices,^1,2^ bio-actuators^3,4^ solar cells,^5,6^ tissue engineering^7,8^ and biosensors.^9,10^ Its advantages, studied so far, include multiple color transitions, depending upon the pH of its synthesis and its oxidation states; its tunable conductivity and maintenance of electrochemical behavior by monitoring the surrounding pH, type of dopant and doping intensity, oxidation state of PANI, its morphology, thickness, design; electrochemical, chemical, and environmental stability due to strong and stable heterocyclic aromatic backbone; ease of processability due to its simple synthetic methodology and its high solubility in innumerable solvents; and capability to fabricate versatile composites, nanocomposites, nano-biocomposites in view of its chemical skeleton rich of functionalities and low cost. These advantages make PANI a material known for wide spectrum of applications. All of these features are related to its chemical structure. Chemically, this polymer comprises of “*n*” number of reduced benzenoid diamine and “*m*” number of oxidized quinoid diamine repeating units, where the oxidation state of polyaniline (PANI) depends on the value of “*m*.” The three different redox forms of PANI are: leucoemeraldine, emeraldine, and pernigraniline the having *m* : *n* ratio of 0 : 1, 1 : 1, and 1 : 0, respectively, Fig. 1. Besides the imine groups, the amine groups in the polymer chains can further be protonated in the presence of H^+^ (acidic) ion to generate cationic defects (polarons and bipolarons) that are responsible for the conductivity and redox behavior of the polymer. The two forms *i.e.* the unprotonated and protonated forms of PANI are known as base and salt, respectively. Conclusively, the conductivity of PANI can be tuned by the use of different doping agents, varying the extent of doping, and also by controlling the chain length and morphology including the porosity and dimensions.

## 2. Synthesis techniques of PANI

PANIs are synthesized using both chemical and electrochemical oxidative polymerization techniques in acidic medium.^11,12^ Usually, the electrochemical method is used for the small scale synthesis, whereas the chemical method allows large-scale preparation of the polymer and/or the corresponding nanocomposites. The electrochemical methods include the electrode coating and co-deposition approaches. In the electrode coating method, reference, working, and counter electrodes are used in a one compartment cell containing the electrolyte and the monomer solution.^13,14^ In the co-deposition method, an insulating polymer host is dissolved in an electrolyte solution comprising the monomer of the conductive polymer. Potentiodynamic and galvanostatic procedures have been used to prepare PANI. The electrochemical method has a number of advantages over the chemical method such as easy approach, attainment of homogeneous films, purity of the end product. In the process of chemical oxidation polymerization, the polymer PANI is synthesized by utilizing HCl/H_2_SO_4_ as a dopant and ammonium persulfate (APS)/FeCl_3_ as an oxidant under aqueous environmental conditions.^15,16^ Afterwards, a proton can be removed by means of an oxidant from the monomer of aniline entity without creating a new bond, and with the absolute product. Ammonium sulfate ((NH_4_)_2_S_2_O_8_), sodium vanadate (NaVO_3_), hydrogen peroxide (H_2_O_2_), potassium dichromate (K_2_Cr_2_O_7_), cerium sulfate (Ce(SO_4_)_2_), potassium free cyanide (K_3_[Fe(CN)_6_] and potassium iodate (KIO_3_) can be used as oxidants.^17,18^

## 3. Applications in protein detection

PANI is highly efficient in detection and sensing due to its redox behaviour and the capacity to mediate electron transfer between the reaction site and the electrode surface *via* biomolecules. The existence of redox couples in PANI facilitates charge transfer activities, making it an excellent option for electrochemical biosensor development.^19–25^ The consistent and sensitive interdependence between the electrochemical response and the pH of the electrolytic solution opens up new possibilities for developing pH sensitive electrochemical biosensors for analytes that generate either acidic or basic moieties as end products of any biochemical reaction, such as triglycerides. The amine enriched chemical backbone of PANI offers a wide range of possibilities for binding/immobilizing biomolecules. Several studies have been reported on the design and fabrication of PANI based protein sensors as summarized in Table 1.

**Table 1:** PANI based protein sensors with their detection limits

Sensor composition	Method of detection	Analyte	Detection limit
Single PANI nanowire^19^	Conductance	Immunoglobulin G (IgG)	3 ng mL^−1^
		Myoglobin (Myo)	1.4 ng mL^−1^
PANI molecularly imprinted polymers (MIPs)^20^	Luminescence quenching	Horseradish peroxidase (HRP)	1.00 on glass slips 0.07 ng mL^−1^ on polycapillaries
Molecular imprinted MIP-PANI^21^	Electrochemical	Ovalbumin (OVA)	10^−12^ mg mL^−1^
Aptamer functionalized PANI nanowire^22^	Electrochemical	Immunoglobulin E (IgE)	0.56 pg mL^−1^ (signal-to-noise ratio of 3)
Whatman filter paper coated with PANI^23^	Impedance	Myoglobin (Myo), Myeloperoxidase (MPO)	500 ng mL^−1^
PANI and electro-deposited gold (Au) nanocrystals (NCs)^24^	Impedance	Human serum albumin (HSA)	300 μg mL^−1^ cell lysate and actin protein
Single site-specific polyaniline (PANI) nanowire^25^	Conductometric sensing	Cardiac troponin-I (cTnI), myoglobin (Myo), creatine kinase-MB (CK-MB), and type-b natriuretic peptide (BNP)	cTnI (250 fg mL^−1^), Myo (100 pg mL^−1^), CK-MB (150 fg mL^−1^), BNP (50 fg mL^−1^)
2-D PANI nanostructures on both non-flexible (SiO_2_) and polyethylene terephthalate and polyimide flexible substrates^26^	Electrochemical	B-type natriuretic peptide (BNP)	100 pg mL^−1^
PANI-Au modified paper working electrodes^27^	Electrochemical	Carcinoembryonic antigen (CEA) and fetoprotein (AFP)	0.5 and 0.8 pg mL^−1^
Electrospun polystyrene (PS)/PANI nanofiber^28^	Electrochemical	C-reactive protein (CRP)	100 fg mL^−1^
PANI electrochemically deposited on Si^29^	Electrochemical	Human IgG antigen	5 μg mL^−1^
Acrylic acid (AAc) is grafted PANI^30^	Surface adsorption studies	ICHA antigen	50 μg mL ICHA concentration surface covering achieved
PANI nanowires^31^	Electrochemical	Immunoglobulin G (IgG)	0.27 ng mL^−1^
MoS_2_–PANI gold nanoparticles^32^	Electrochemical	C-reactive protein (CRP)	40 pg mL^−1^
PANI films^33^	Electrochemical	Cholesterol oxidase (CHOx), cholesterol esterase (ChEt)	25 mg dL^−1^
Nanostructured PANI sheets^34^	Electrochemical	Cholesterol oxidase	25–500 mg dL^−1^
PANI nanospheres^35^	Electrochemical	Cholesterol oxidase	25–500 mg dL^−1^
Polyaniline nanospheres^36^	Electrochemical	DENV NS1 antibody	0.33 ng mL^−1^
PANI film^38^	Electrochemical	Cholesterol oxidase, cholesterol peroxidase, and cholesterol esterase	25 mg dL^−1^
PANI nanowire and hyaluronic acid^39^	Electrochemical	Carcinoembryonic antigen (CEA)	0.0075 pg mL^−1^
PANI–Au nanocomposite^40^	Electrochemical	Cholesterol oxidase	37.89 mg dL^−1^
PANI and carboxylated PANI on graphene oxide (GO)^41^	Electrochemical	cTnT for cardiac troponin T probe	0.008 ng mL^−1^
Manganese sulfide nanoparticles/graphene oxide/polyaniline (MnS/GO/PANI) and magnetite incorporated gold nanoparticles (AuNPs@Fe_3_O_4_)^42^	Electrochemical	Tau protein	10^−14^ M
PANI/CNT^43^	Electrochemical	Vascular endothelial growth factor (VEGF165)	0.4 pg mL^−1^
PANI nanowires^44^	Electrochemical	COVID-19 N-gene	3.5 fM

**Fig. 2:**
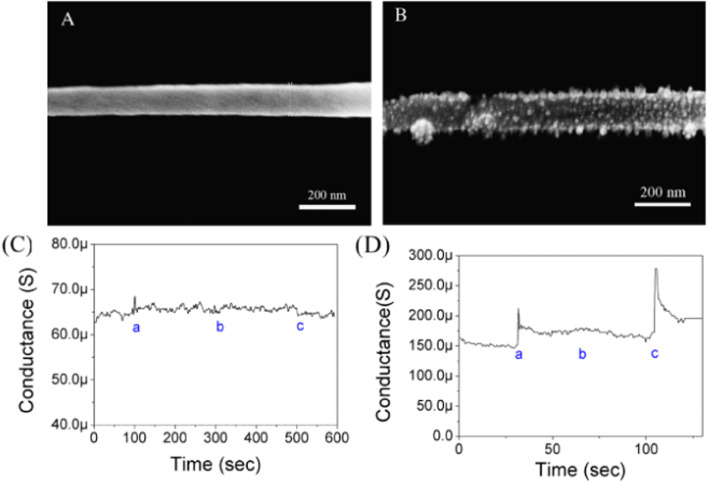
(A) SEM image of non-functionalized single PANI nanowire. (B) SEM image of functionalized single PANI nanowire with IgG mAbs. (C) Measurement of conductance changes on single non-functionalized PANI nanowire (a: PBS; b: BSA, 10 μg mL^−1^; and c: IgG, 3.3 μg mL^−1^). (D) Specificity test of functionalized single PANI nanowire with IgG mAbs (a: PBS; b: BSA, 50 ng mL^−1^; and c: IgG, 33 ng mL^−1^) (reprinted with permission from Elsevier, ref. 19 Lee *et al.*, (2011)).

Proteins can be immobilized showed that the detection/sensing of proteins like immunoglobulin G (IgG) and myoglobin (Myo) is based on measuring the conductance changes of PANI in response to target proteins and their various concentrations, Fig. 2.

The Fig. 2(A) shows non-functionalized single PANI of width 105.4 nm while the Fig. 2(B) revealed the deposition of few particles ranging between 10 nm to 15 nm after functionalization of PANI nanowire which were speculated to be immobilized IgG mAbs. The IgG protein was examined and the feasibility of a single PANI nanowire biosensor was investigated *via* with a conductometric mechanism, Fig. 2(C) and (D). There were no changes in the non-functionalized PANI nanowire biosensor. The conductance of the functionalized single PANI nanowire biosensor with IgG towards monoclonal antibodies (mAbs) increased with PBS injection due to a change in the surface charge generated by the net electrical field. The unique IgG (33 ng mL^−1^) response after BSA injection, demonstrated the biosensor's high sensitivity for IgG detection. IgG mAbs exhibited stepwise changes upon varying the concentrations of IgG. The lowest limit of detection was 3 ng mL^−1^ for IgG and 1.4 ng mL^−1^ for Myo, while a clear non-response towards BSA was observed at concentrations as high as 5 μg mL^−1^.

**Fig. 3:**
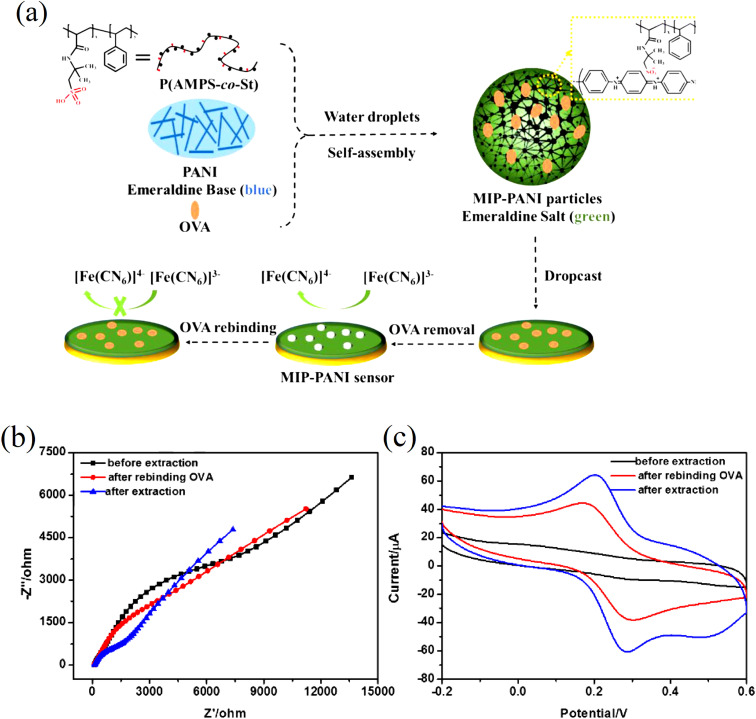
(a) Schematic illustration of the synthesis of MIP-PANI particles and the fabrication of MIP-PANI sensor, (b) EIS curves and (c) CV curves of MIP-PANI particles modified electrode in [Fe(CN)6]^3−/4−^ solution before extraction, after extraction and after rebinding OVA by incubation in 0.5 mg mL^−1^ OVA solution (reprinted with permission from Elsevier, ref. 21 Luo *et al.*, (2017)).

Pidenko *et al.*^20^ proposed a facile and fast approach for the synthesis of PANI molecularly imprinted polymers (MIPs) based on oxidative chemical polymerization of aniline monomers that were used for protein recognition. It was for the first time that the strategy of surface imprinting was implemented for the synthesis of PANI-MIPs on the inner surface of glass polycapillaries (PC) having 2237 number of microcapillaries. Uniform and stable PANI films were synthesized by means of oxidative polymerization at around pH less than 1. PANI MIP nanowires were however synthesized at “milder” conditions (pH greater than 4.5) to keep the protein template activity. The binding ability of horseradish peroxidase (HRP) molecules on the as-synthesized PANI-MIP selective sites was confirmed by means of photometry, FTIR spectroscopy and SEM images. The as developed PANI-MIPs enabled HRP determination with low limit of detection (LOD) *i.e.* 1.00 on glass slips and 0.07 ng mL^−1^ on PC. Luo *et al.*^21^ used ovalbumin (OVA) as a template protein which was trapped inside the PANI nanoparticles (NPs), resulting in the production of molecular imprinted MIP-PANI, Fig. 3(a).

**Fig. 4:**
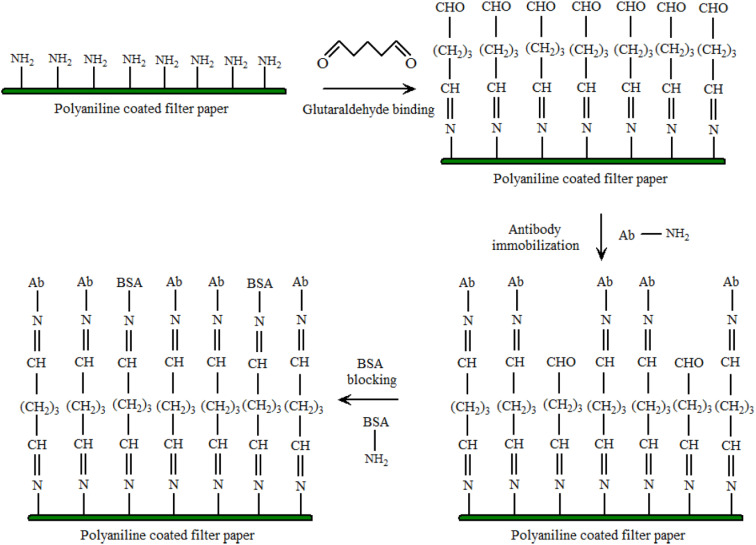
Functionalization of PANI coated filter paper. The amine groups of PAni bonds with the one of the two aldehyde (–CHO) groups of Glut. The other –CHO group is available for covalent bonding with the amine groups of the antibodies. A wash step is present between each modification step (reprinted with permission from IEEE, ref. 23 Mondal *et al.*, (2017)).

**Fig. 5:**
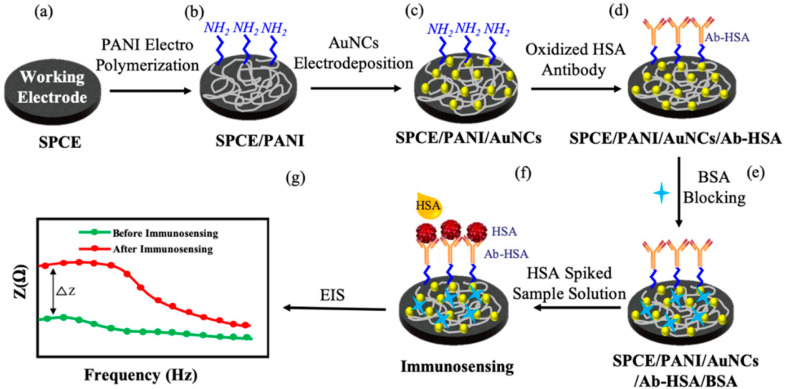
Schematic of the systematic protocol for SPCE surface modification and immunosensing. PANI – polyaniline; AuNCs – gold nanocrystals; HSA – human serum albumin; Ab-HSA – anti-human, serum albumin antibody; BSA – bovine serum albumin; EIS – electrochemical impedance spectroscopy (reprinted with permission from MDPI, ref. 24 Shaikh *et al.*, (2019)).

The PANI sensor not only showed good selectivity towards template protein (the imprinting factor was 5.31), but also significantly lower detection limit of 10^−12^ mg mL^−1^, outperforming most of the reported OVA detecting methods. Furthermore, a response time of less than 3 minutes was demonstrated. The water compatibility as well as large specific surface area of PANI particles, and electrical conductivity of PANI, provided a direct channel for electron conduction from the imprinting sites to the electrode surface, Fig. 3(b) and (c). Based on the immobilisation of the immunoglobulin-E (IgE) aptamer onto a single PANI nanowire electrochemically produced in a facile and controllable method, Luo *et al.*^22^ synthesized a highly sensitive, conductometric, and label-free biosensor for the detection of IgE. Myoglobin (Myo) and Myeloperoxidase (MPO) cardiac biomarkers were detected using a disposable, label-free impedance-based biosensor. Mondal *et al.*^23^ fabricated a biosensor out of Whatman filter paper that was coated with PANI, Fig. 4. Prior to the covalent attachment of antibodies unique to each biomarker protein, the PANI-coated paper was functionalized with glutaraldehyde. Treatment with BSA blocked the non-specific active areas of the sensor. Myo and MPO in buffer solution were detected at concentrations ranging between 100 ng mL^−1^ and 50 g mL^−1^. For these cardiac biomarkers spiked in human serum, the detection limit was 1 g mL^−1^. The lowest detection limit was improved to 500 ng mL^−1^ after further blocking with human serum albumin (HSA). The developed biosensor was appropriate for diagnostic applications due to its low-cost fabrication procedure, detection of cardiac biomarkers in clinically relevant quantities quickly (*i.e.*, within 20 minutes), and environmentally acceptable disposable nature. The presence of minute levels of HSA in urine, also known as microalbuminuria (30–300 g mL^−1^) was utilized as a useful clinical diagnostic method used for detecting chronic kidney disease (CKD). Shiakh *et al.*^24^ provided a report in which they had used electrochemical impedance spectroscopy for the creation of a low-cost, disposable immunosensor for the specific, sensitive, and label-free detection of HSA, Fig. 5.

**Fig. 6:**
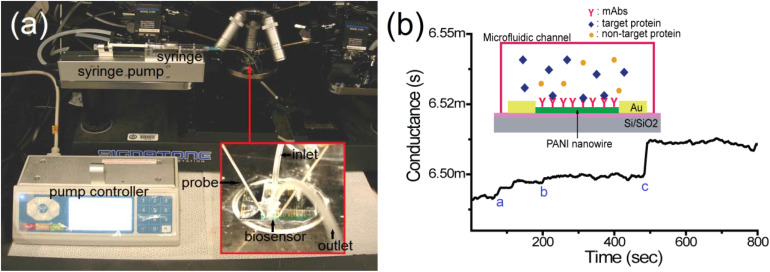
An illustration and the experimental set-up of the single polyaniline (PANI) nanowire biosensor to detect cardiac biomarkers. (a) The experimental setup; the microfluidic channel is adhered on the nanowire biosensor and the nanowire biosensor chip is mounted on a probe station connected to the semiconductor analyzer and syringe pump with inlet and outlet; (b) the conductance change in the single PANI nanowire-based biosensor is monitored. The injection of PBS (mark a), BSA (mark b), and cardiac biomarker (mark c) shows the different changes of conductance (reprinted with permission from MDPI, ref. 25 Lee *et al.*, (2017)).

In order to develop the carbon-based tri-electrode system on some flexible plastic substrates, a straightforward one-step screen-printing process was utilized. The carbon working electrode was later on modified with some electro-polymerized PANI and electro-deposited gold (Au) nanocrystals (NCs) to enable an effective antibody immobilization and increased sensitivity. The PANI matrix functioned as a nanostructured scaffold for uniform distribution of the Au NCs, resulting in an increased immunosensor response. SEM was used to study the PANI/Au NCs-modified working electrode surface, and electrochemical impedance spectroscopy (EIS) was used to investigate the electrochemical response at every step in a probe solution of ferri/ferrocyanide. The immunosensor showed good selectivity as normalized impedance variation of 3.6% ± 0.6% and 4.7% ± 1% was observed for immunosensing with 300 μg mL^−1^ cell lysate and actin protein, and was lower for the same concentration of HSA. In order to diagnose the cardiovascular disorders, proteins like cardiac troponin-I (cTnI), myoglobin (Myo), creatine kinase-MB (CK-MB), and type-b natriuretic peptide (BNP) must be detected. Lee *et al.*^25^ showed that single-site-specific PANI nanowire biosensors had ultra-high sensitivity and specificity for cardiac biomarkers, Fig. 6. Very low amounts of Myo (100 pg mL^−1^), cTnI (250 fg mL^−1^), CK-MB (150 fg mL^−1^), and BNP (50 fg mL^−1^) were detected using single PANI nanowire-based biosensors connected with microfluidic channels. For concentrations ranging from a few hundreds (fg mL^−1^) to tens of hundreds (ng mL^−1^), the single PANI nanowire-based biosensors showed linear sensing characteristics.

In addition to that, the devices demonstrated a quick response of minutes that met the reference conditions for Myo, CK-MB, BNP and cTnI diagnosis of heart failure, as well as determining the severity of heart failure. PANI also showed strong biocompatibility with monoclonal antibodies, due to which its nanowire-based biosensor displayed superior biosensing reliability, label-free detection, and highly improved processing charge efficiency.

Liu *et al.*,^26^ designed field-effect transistor (FET) biosensors based on two-dimensional (2-D) PANI nanostructures on both non-flexible (SiO_2_) and polyethylene terephthalate and polyimide flexible substrates. The biosensors were made using a simple and low-cost technology that combined top-down and bottom-up methods. The development of a low-temperature bilayer method greatly enhanced the yield of flexible electronics. Chemically produced PANI nanostructures demonstrated outstanding p-type semiconductor characteristics as well as flexibility in design. The produced biosensors displayed remarkable sensing capability in detecting B-type natriuretic peptide (BNP) biomarkers, with the 2-D PANI nanostructure being as thin as 80 nm and an extraordinarily large surface-area-to-volume (SA/V) ratio due to the intrinsic features of PANI chemical production. Both designs showed excellent repeatability and high specificity, with detection limits as low as 100 pg mL^−1^. The controllable conductance fluctuations of less than 20% with good restorability of the PANI nanostructure under bending conditions were also examined. Li *et al.*^27^ carried out the simultaneous measurement of two tumour markers, carcinoembryonic antigen (CEA) and α-fetoprotein (AFP), in actual human serum samples using PANI-AuNP-modified paper working electrodes (PWEs). PANI/Au-PWE exhibited good biocompatibility and retained good stability for the sandwich-type immunoassay. The multiplex immunoassay showed linear ranges of over 4 orders of magnitude with the detection limits being down to 0.5 and 0.8 pg mL^−1^ respectively for CEA and α-fetoprotein (AFP).

**Fig. 7:**
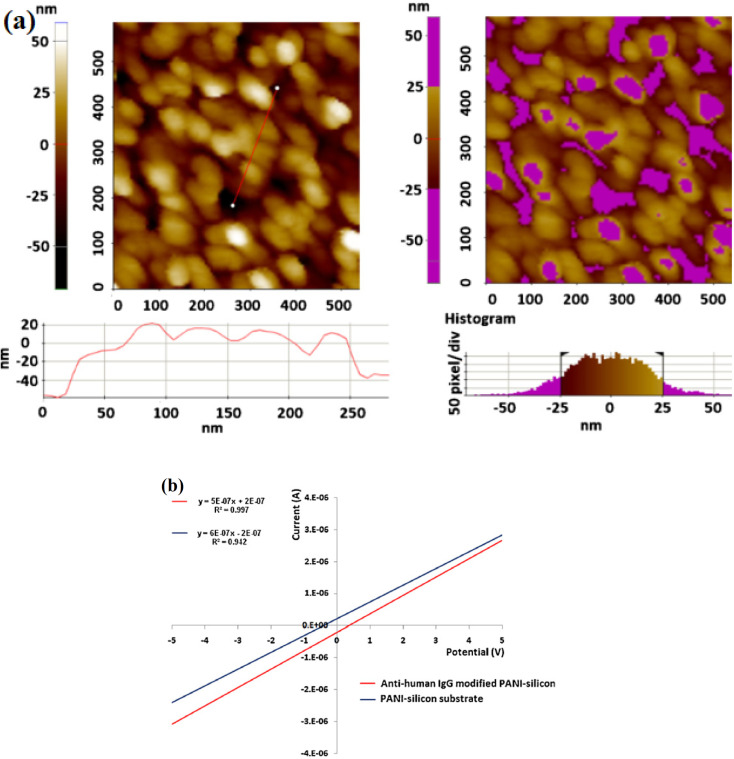
(a) AFM topography of the electrodeposited PANi film on Si surface; line (left image) and region (right image) analysis to estimate the average height of the deposited film. Data suggest the formation of thin film of 45–50 nm with globular PANi structures, (b) current–voltage characteristic (regression data) of the PAni–Si substrate and the formed biosensor ‘anti-human IgG–PAni–Si’. Ohmic conduction characteristics are evident with average conductivities of 45 μS for the PAni–Si substrate and 32 μS for the formed bioelectrode (reprinted with permission from Elsevier, ref. 29 Deep *et al.*, (2012)).

Anwar *et al.*^28^ designed a “label-free” biosensor for detecting the cardiovascular risk biomarker C-reactive protein (CRP). An electrospun polystyrene (PS)/PANI nanofiber was used as the biosensor's sensing element, which was coupled to an electrical measurement apparatus. Size-matched confinement for micro biomolecule immobilization was possible using nanoscale fibers with a high aspect ratio and porosity, resulting in faster transmission and higher signal intensity. At a concentration of 100 fg mL^−1^, CRP was successfully detected in human serum, demonstrating that this lab-on-chip technique could be used to detect cardiovascular risk early. Less than 10% cross-reactivity with human albumin demonstrates the suggested biosensor's selective detection capabilities. PANI was electrochemically deposited on a silicon (Si) substrate by Deep *et al.*^29^ to form thin nanostructured films with a thickness of around 50 nm. These films were coated with avidin and subsequently immobilised with biotinylated anti-human IgG to generate a sensing bioelectrode. The deposited PANI thin layers on the Si substrate were examined using atomic force microscopy (AFM), Fig. 7(a).

AFM revealed globular PAni nanostructures and the film thickness was investigated to be around 45–50 nm. Growth of PAni as globular nanostructures on the p-Si(1 1 1) wafers absorbed atmospheric O_2_ that caused an inhomogeneous thin oxide layer with number of defects serving as the active sites for the assembly of PANI in nanostructured pattern. The biomolecules were successfully immobilized, according to Raman spectrometric studies. The current–voltage characteristics of the built sensor were utilized to calculate the concentration of human IgG antigen in the range of 5–550 μg mL^−1^, Fig. 7(b). The suggested biosensor's detection limit and sensitivity are 5 μg mL^−1^ and 0.15 S ppm^−1^ human IgG, respectively.

**Fig. 8:**
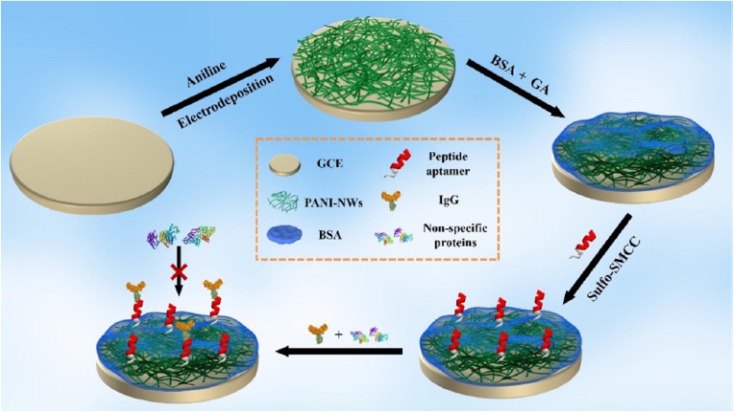
Schematic illustration of the fabrication process of the electrochemical IgG biosensor based on a BSA/PANI-NW antifouling interface (reprinted with permission from American Chemical Society, ref. 31 Li *et al.*, (2021)).

Crombrugghe^30^ synthesized PANI grafted transducer. Bioreceptors were covalently bonded to the polymeric surface to achieve selective and efficient bioanalyte detection. Using typical coupling agents, carboxylic groups can be used to covalently immobilise proteins like the ICHA antigen (carbodiimide and succinimide). A surface covering of 50 μg mL^−1^ ICHA concentration, was attained which did not form a compact monolayer. Li *et al.*^31^ fabricated a highly sensitive and antifouling biosensing interface based on the completely cost-effective and inert protein bovine serum albumin (BSA) cross-linked with PANI nanowires (NWs), Fig. 8.

The cross-linking of BSA demonstrated a relatively significant antifouling capacity when compared to physically adsorbed BSA, which was widely utilized to inhibit nonspecific adsorption/binding of proteins. An electrochemical biosensor with outstanding sensitivity and selectivity was produced using a suitable immobilisation of the peptide aptamer for protein immunoglobulin G (IgG) recognition onto the BSA in PANI-NW interface. The IgG biosensor that was developed had a linear range of 1.0 ng mL^−1^ to 10 g mL^−1^ and a very low detection limit of 0.27 ng mL^−1^, and was capable of accurately assaying IgG in complex human serum samples. The IgG biosensor showed a linear range of 1.0 ng mL^−1^ to 10 g mL^−1^ and a low detection limit of 0.27 ng mL^−1^ as compared to the findings obtained using commercial enzyme-linked immunosorbent test kits.

Molybdenum sulphide PANI gold nanoparticles (MoS_2_–PANI–GNPs) with a large surface area and many adsorbing sites significantly increased antibody loading amounts and hence improved biosensing performance. The proposed immunosensor was used to determine C-reactive protein (CRP) in HSA with success.^32^ The sensing technique had a linear dynamic range of 0.2 to 80 ng mL^−1^ and a detection limit of 40 pg mL^−1^. Singh *et al.*^33^ used cholesterol oxidase (CHOx), cholesterol esterase (ChEt), and HRP enzymes to modify electrochemically produced PANI films and examine their optical and electrochemical biosensing response to total cholesterol. PANI-based cholesterol oxidase/cholesterol esterase films showed a detection limit of 25 mg dL^−1^ with sensitivity of 0.042 μA mg dL^−1^. Dhand *et al.*^34^ proposed the use of electrophoretically deposited nanostructured PANI sheets in the production of cholesterol biosensors. For assessing the photometric response of the created bioelectrodes, *o*-dianisidine was used. Electrophoretically synthesized PANI matrices improved cholesterol oxidase–cholesterol interactions. The PANI based bioelectrode detected cholesterol in a wide range of 25–500 mg dL^−1^ and a high sensitivity of 3.57 × 10^−4^ mA mg dL^−1^ was attained with a low response time of 30 s high electroactive behaviour of PANI matrix. PANI nanospheres (NS) were manufactured utilizing an ethylene glycol-mediated morphological transformation approach and investigated for cholesterol biosensing in another interesting research by Dhand *et al.*^35^ The nanospheres film was deposited onto an indium-tin-oxide (ITO) coated glass plate by the solution casting method and was utilized for covalent immobilization of cholesterol oxidase (ChOx) *via N*-ethyl-*N′*-(3-dimethylaminopropyl) carbodiimide (EDC) and Nhydroxysuccinimide (NHS). The ChOx/PANI-NS/ITO bioelectrode was able to detect cholesterol in the concentration range of 25 to 500 mg dL^−1^ with sensitivity of 1.3 × 10^−3^ mA mg dL^−1^.

**Fig. 9:**
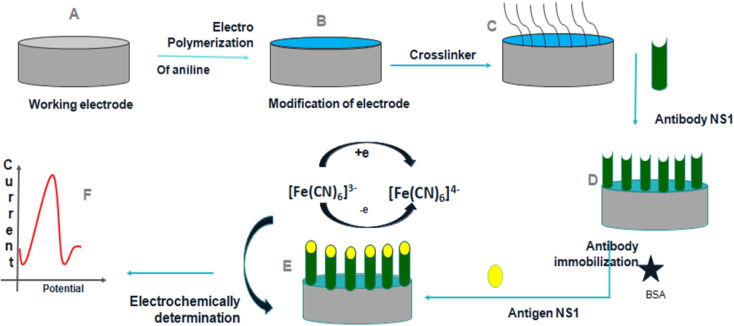
Schematic representation of construction and development of the immunosensor (reprinted with permission from Avicenna Journal of Medical Biotechnology, ref. 36 Dutta *et al.*, (2020)).

Dutta *et al.*^36^ suggested a label-free electrochemical immunosensor based on a PANI-modified GC electrode for the detection of dengue NS1 protein, Fig. 9. The accuracy of NS1 determination was tested using an ELISA dengue analysis kit to compare the results obtained from the immunosensor. The immunosensor detected in-house expressed, commercially acquired, spiked, and blood NS1 samples, demonstrating its sensitivity across a diverse set of samples. The limit of detection was investigated to be 0.33 ng mL^−1^.

Langer *et al.*^37^ used PANI layers with regulated porosity to immobilise choline oxidase in a choline sensor. The obtained sensor was found to be extremely stable, and the electrical response of the sensor stayed constant for more than a month. The electrical response was found to be dependent on choline concentration showing a sensitivity of 5 μA mM^−1^ in the amperometric mode and of 10 mV mM^−1^ in the potentiometric mode of measurements.

**Fig. 10:**
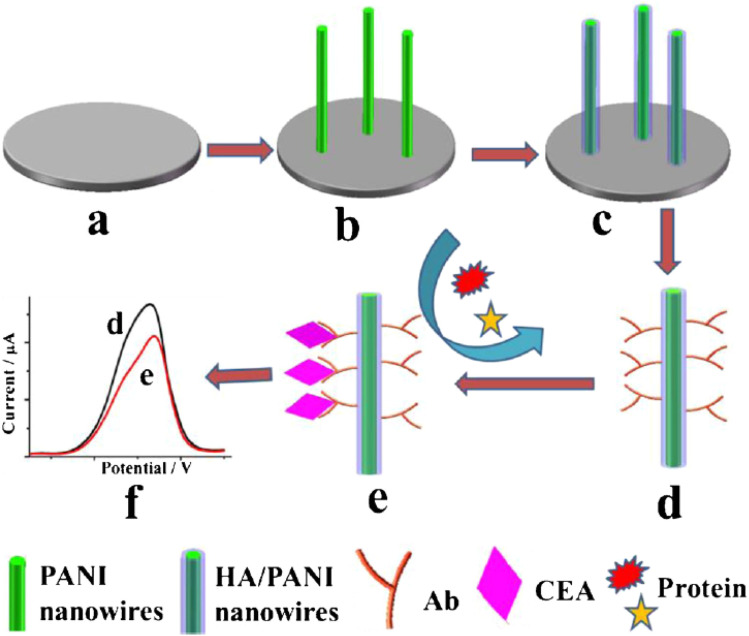
Stepwise fabrication process of the immunosensor. (a) The bare GCE, (b) PANI nanowires electrodeposited on the GCE, (c) the formation of HA/PANI composite wires, (d) CEA antibody immobilization, (e) specific CEA capturing and the antifouling towards nonspecific protein and (f) DPV current signal recording (reprinted with permission from Springer, ref. 39 Wang *et al.*, (2018)).

Singh *et al.*^38^ suggested a biosensor for determining cholesterol levels that uses electrochemically produced PANI-enzyme film. Cholesterol oxidase, cholesterol peroxidase, and cholesterol esterase were all co-immobilized to create these films. The biosensor had a 240-second response time, a 42-day shelf life, a sensitivity of 0.042 A mg dL^−1^, and a detection limit of 25 mg dL^−1^. Wang and his colleagues^39^ created a non-fouling electrochemical immunosensor using a hybrid structure made of PANI nanowire and hyaluronic acid (HA) which was then immobilized with carcinoembryonic antigen (CEA) antibodies, Fig. 10.

**Fig. 11:**
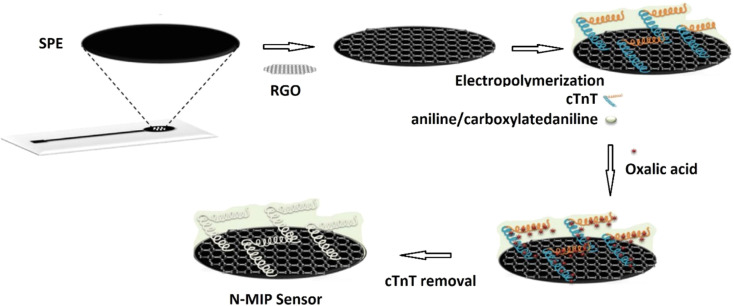
Schematic of the N-MIP sensor (reprinted with permission from Elsevier, ref. 41 Karimi *et al.*, (2019)).

The antifouling efficacy of the PANI/HA surface was evaluated and the detection limit of the immunoelectrode was 0.0075 pg mL^−1^, with a linear response of 0.01 to 10 000 pg mL^−1^. Srivastava *et al.*^40^ used chitosan (CH) and PANI–Au nanocomposite to manufacture a biosensor with good sensitivity and accuracy for cholesterol detection. Chemically produced PANI/Au nanocomposite was used as a matrix for immobilisation of cholesterol oxidase (ChOx) enzyme on the modified electrode surface, together with CH. The linearity of this enzymatic biosensor was 50–500 mg dL^−1^, with a detection limit of 37.89 mg dL^−1^ and a sensitivity of 0.86 mA mg dL^−1^. It was inferred that this matrix outperforms other cholesterol sensors due to its good immobilisation and enzyme–substrate reactions. Karimi *et al.*^41^ created a biomimetic nano-molecularly imprinted polymer (N-MIP) electrode for the ultrasensitive detection of cardiac troponin T based on a graphene screen-printed electrode (cTnT). By electropolymerizing conductive co-polymer matrix of aniline and carboxylated aniline on the graphene oxide (GO) electrode in the presence of template protein (cTnT for cardiac troponin T probe) and cyclic voltammetry (CV), biomimetic cavities for targeted analyte sensing were produced, Fig. 11. The detection limit of the cTnT probe was 0.008 ng mL^−1^, with a linear range of 0.02 to 0.09 ng mL^−1^. Lately a tau protein immunosensor based on manganese sulphide (MS) graphene oxide (GO) and PANI (MnS/GO/PANI) with magnetite-incorporated gold nanoparticles immunosensor has been developed for the electrochemical detection of tau protein which exhibited superior selectivity, sensitivity, and environmental compatibility.^42^ The sensor demonstrated the a detection limit of 1.0 × 10^−14^ M which could be utilized for diagnosis of Alzheimers disease. Park *et al.*^43^ designed an aptasensor based on PANI and carbon nanotube (CNT) for detecting VEGF165 as a tumor marker with a limit of detection of 0.4 pg mL^−1^ and showed high selectivity in presence of other proteins. Song *et al.*^44^ formulated PANI nanowires immobilized with an inverted Y-shaped peptide as an antifouling agent and the specific N-gene capture probe (an antisense oligonucleotide) as a recognition element for the detection of the COVID-19 N-gene (nucleocapsid phosphoprotein gene). The detection limit for the COVID-19 N-gene, reached as low as 3.5 fM even in complex human serum samples which showed immense potential for early detection of COVID-19 viral infection.

## 4. Conclusion and future aspects

PANI-based biosensors have received special attention during the past several decades. Different nanostructured PANI composites, PANI 2D films, and PANI nanowires have been investigated and have shown immense selectivity as well as sensitivity towards a variety of protein analytes. PANI based selective probes, including metal oxides and carbon nanotubes have been developed for efficient biosensing platform. Nanostructured PANI shows increased sensitivity and faster response time due to changes in the electrostatic charges from surface adsorption of various protein molecules, which causes accumulation/depletion of charge carriers in PANI. The functionalization of the PANI nanowires could be utilized to improve resolution and good specificity of target detection. The construction of sensing interfaces with protein-cross-linked conducting polymers can be extended for the development of biosensors with high selectivity for assaying of different targets in biological media.

## Conflicts of interest

There are no conflicts to declare.

## Supplementary Material
